# Plasma angiopoietin-like 4 is related to phospholipid transfer protein activity in diabetic and non-diabetic subjects: role of enhanced low grade inflammation

**DOI:** 10.1186/s12944-018-0717-5

**Published:** 2018-03-27

**Authors:** Eke G. Gruppen, Sander Kersten, Robin P. F. Dullaart

**Affiliations:** 1 Department of Endocrinology, University of Groningen and University Medical Center, P.O. Box 301, 9700 RB Groningen, The Netherlands; 2 Division of Human Nutrition, Wageningen University, Wageningen, The Netherlands

**Keywords:** Angiopoietin-like4, High sensitivity C-reactive protein, Non-esterified fatty acids, Phospholipid transfer protein activity, Type 2 diabetes mellitus

## Abstract

**Background:**

Angiopoietin-like 4 (ANGPTL4) inhibits lipoprotein lipase, whereas phospholipid transfer protein (PLTP) enhances hepatic triglyceride secretion. Both factors may be upregulated by inflammatory pathways. Since the extent to which these circulating factors are interrelated is unknown, we determined the relationship between plasma ANGPTL4 and PLTP activity, and assessed whether such a relationship could be explained by high sensitivity C-reactive protein (hsCRP) levels as a marker of low-grade chronic inflammation.

**Methods:**

Fasting plasma ANGPTL4, PLTP activity (liposome-vesicle high density lipoprotein system) and hsCRP were measured in 41 type 2 diabetic (T2DM) subjects and 36 non-diabetic subjects.

**Results:**

Plasma ANGPTL4 and PLTP activity were increased in T2DM (*p* < 0.001 for each), coinciding with elevated hsCRP, triglycerides and non-esterified fatty acids (NEFA) (*p* = 0.031 to 0.001). In univariate analysis, ANGTLP4 was correlated with PLTP activity (Rs = 0.309, *p* = 0.006), whereas both factors were related to hsCRP and NEFA levels (Rs = 0.304 to 0.411, *p* < 0.01 to < 0.001). In multivariable linear regression analysis adjusting for age, sex, glucose, total cholesterol, triglycerides and NEFA, ANGPTL4 and PLTP activity each remained positively associated with hsCRP (β = 0.315, *p* = 0.003 and β = 0.299, *p* = 0.034, respectively). Plasma ANGPTL4 remained positively associated with PLTP activity when taking account of age, sex, glucose, total cholesterol, triglycerides and NEFA (β = 0.315, *p* = 0.003). Notably, this association disappeared after further adjustment for hsCRP (β = 0.131, *p* = 0.25).

**Conclusions:**

In conclusion, plasma ANGPTL4 and PLTP activity are interrelated, which may at least in part be explained by low-grade chronic inflammation. A pro-inflammatory state could affect triglyceride metabolism via concerted effects on ANGPTL4 and PLTP.

## Background

During the past few years, the key role of angiopoietin like proteins (ANGPTL) in lipid metabolism and energy balance is increasingly appreciated [1–3]. Among this family of proteins, ANGPTL4 is a secreted glycoprotein that is highly expressed in liver and adipose tissue [1–3]. Evidence abounds that ANGPTL4 functions as an inhibitor of lipoprotein lipase, thereby attenuating the uptake of triglyceride-rich lipoprotein-derived fatty acids in several cell types including adipocytes [2]. Importantly, non-esterified fatty acids (NEFA) serve as potent activators of ANGPTL4 expression in several tissues, which is mediated by peroxisome proliferator-activated receptors (PPARs) [2, 4–7]. Consistent with these data, circulating ANGPTL4 concentrations have been reported to correlate positively with plasma NEFA, although this relationship has not been consistently observed, while a relationship with fasting triglycerides appears to be modest or even absent [8–10].

The metabolism of triglycerides is also governed by phospholipid transfer protein (PLTP), a lipid transfer protein that is produced by a variety of cell systems, including hepatocytes, adipocytes and macrophages [11–13]. PLTP transfers phospholipids between lipoproteins, and converts high density lipoproteins (HDL) to smaller and larger particles [11–13]. Of note, studies in transgenic mice have shown that PLTP deficiency attenuates the secretion of very low density lipoproteins (VLDL) by the liver, whereas PLTP overexpression has the opposite effect [14, 15]. Moreover, studies in humans have shown that plasma PLTP activity is increased after a prolonged exposure to an intravenously administered triglyceride emulsion, while a decrease has been observed when plasma NEFA availability is attenuated by Acipimox administration [16, 17].

Interestingly, recent evidence has linked ANGPTL4 regulation to inflammatory pathways. Specifically, treatment of mice with lipopolysaccharide or zymosan was shown to increase ANGPTL4 expression in several tissues [18]. Furthermore, Angptl4 mRNA and protein levels are upregulated by various inflammatory stimuli in human THP1 macrophages in vitro [10]. Clinically, plasma ANGPTL4 correlates positively with high sensitivity C-reactive protein (hsCRP), an established marker of enhanced low-grade inflammation [8, 10, 19]. Accordingly, the plasma ANGPTL4 concentration is likely to be elevated in subjects with pro-inflammatory conditions such as the metabolic syndrome and Type 2 diabetes mellitus (T2DM) [9, 10, 20]. Notably, plasma PLTP activity is associated with enhanced low-grade chronic inflammation as well, as evidenced by its positive relationship with hsCRP [21–23]. In keeping with a role of inflammation in the regulation of this lipid transfer protein, plasma PLTP activity is elevated during the acute phase of sepsis in humans [24]. Taken together, these data [10, 21–23] support the hypothesis that enhanced low-grade inflammation, as reflected by plasma hsCRP, may result in upregulation of both circulating ANGPTL4 and PLTP activity. Pathophysiologically, such a relationship between ANGPTL4 and PLTP would conceivably link a pro-inflammatory state to alterations in triglyceride metabolism. Since diabetic dyslipidemia is featured by abnormalities in triglyceride metabolism [10, 11], it is relevant to study ANGPTL4 and PLTP activity together in T2DM.

In the absence of any data concerning the possible relationship between circulating ANGPTL4 levels and PLTP activity, we initiated the present study to determine i) the extent to which plasma ANGPTL4 relates to PLTP activity among subjects with and without T2DM, and ii) whether such a relationship may be explained at least in part by an association of low-grade chronic inflammation, using hsCRP as marker, with ANGPTL4 and PLTP.

## Methods

### Subjects

The study protocol was approved by the medical ethics committee of the University Medical Center Groningen, and written informed consent had been obtained from all participants. Subjects with and without T2DM were aged > 18 years, and were recruited by advertisement in local newspapers. T2DM had been diagnosed by primary care physicians applying guidelines from the Dutch College of General Practitioners (fasting plasma glucose ≥ 7.0 mmol/l; non-fasting plasma glucose ≥ 11.1 mmol/l). The use of metformin, sulfonylurea was allowed but insulin use was an exclusion criterion. Subjects taking antihypertensive medication were also allowed to participate. Subjects with a history of cardiovascular disease, chronic kidney disease (estimated glomerular filtration rate < 60 ml/min/1.73 m^2^ and/or proteinuria), abnormal liver function tests (transaminases > 3 times the upper reference limit) or thyroid dysfunction (thyroid stimulating hormone > 10 or < 0.40 mU/l or use of thyroid function influencing medication), as well as current smokers and subjects who used lipid lowering drugs were also excluded.

Blood pressure was measured after 15 min rest at the left arm in sitting position using a sphygmomanometer. Body mass index (BMI in kg/m^2^) was calculated as weight divided by height squared. The participants were studied after an overnight fast.

### Laboratory analyses

Serum and EDTA-anticoagulated plasma samples were stored at − 80 °C until analysis. Plasma glucose was measured shortly after blood collection with an APEC glucose analyzer (APEC Inc., Danvers, MA, USA). Plasma NEFA were measured with a kit from Wako Diagnostics (HR Series NEFA-HR(2), Wako Chemicals GmbH, Neuss, Germany). Plasma total cholesterol and triglycerides were measured by routine enzymatic methods (Roche/Hitachi cat. Nos 11,875,540 and 1,187,602, respectively; Roche Diagnostics GmBH, Mannheim, Germany). HDL cholesterol was assayed by a homogeneous enzymatic colorimetric test (Roche/Hitachi, cat.no 04713214). Non-HDL cholesterol was calculated as the difference between total cholesterol and HDL cholesterol. Low density lipoprotein (LDL) cholesterol was calculated by the Friedewald formula in case of triglycerides < 4.5 mmol/l. Apolipoprotein A-I (apoA-I) and apolipoprotein B (apoB) were measured by immunoturbidimetry (Roche/Cobas Integra Tinaquant cat no. 03032566, Roche Diagnostics). hsCRP was assayed by nephelometry with a lower limit of 0.175 mg/l (BNII N; Dade Behring, Marburg, Germany). HbA1c was measured by high-performance liquid chromatography (Bio-Rad, Veenendaal, the Netherlands; normal range: 27–43 mmol/mol).

Plasma ANGPTL4 was assayed by enzyme-linked immunosorbent assay (ELISA) as described [5, 10]. In brief, 96-well plates were coated with anti-human ANGPLT4 polyclonal goat IgG antibody (AF3485, R&D systems Netherlands, Abingdon, Oxon, UK) and incubated overnight at 4 °C. After 4 washes with 300 μL phosphate buffered saline (PBS)-Tween20 0.1%, 300 μl of blocking solution (PBS containing 1% bovine serum albumin) was added per well and left for 1 h at room temperature under gentle agitation. 100 μl of 20-fold diluted human plasma was applied to each well, followed by 2 h incubation at room temperature under gentle agitation. A standard curve was prepared with recombinant human ANGPTL4 (3485-AN, R&D systems). This procedure was followed by adding 100 μL of diluted biotinylated anti-human ANGPTL4 polyclonal goat IGG antibody (BAF3485, R&D systems) for 2 h, followed by addition of streptavidin-conjugated horseradish peroxidase for 20 min, and tetramethyl benzidine substrate reagent for 6 min. The reaction was stopped by adding 50 μl of 10% H_2_SO_4_, and the absorbance was measured at 450 nm. The intra-assay coefficient of variation (CV) is 7%. Plasma PLTP activity was assayed with a phospholipid vesicles-HDL system, using [^14^C]-labeled dipalmitoyl phosphatidylcholine as described [25, 26]. The phospholipid transfer promoting properties of cholesteryl ester transfer protein do not interfere with this excess exogenous substrate assay. The method is specific for PLTP activity, and represents the level of active PLTP [25]. Plasma PLTP activity (expressed in arbitrary units (AU); 100 AU corresponds to 13.6 μmol phosphatidylcholine transferred per mL plasma per hour) was related to its activity in reference human pool plasma, and was measured in duplicate. The inter-assay CV amounts to 5%.

### Statistical analysis

IBM SPSS software (SPSS, version 22.0, SPSS Inc. Chicago, IL, USA) was used for data analysis. Data are expressed in medians (interquartile ranges). Between group differences in variables were determined by Mann-Whitney U tests and Chi-square tests where appropriate. Univariate correlations were determined by Spearman correlation coefficients. Multivariable linear regression analyses were carried out to disclose the independent relationships of ANGPTL4 and PLTP activity separately with hsCRP taking account of glycemia, NEFA and lipid levels. Additional analysis were done to discern the influence of hsCRP on the relationship between ANGPTL4 and PLTP activity. In these analyses, ANGPTL4, triglycerides and hsCRP were log_e_ transformed to achieve approximately normal distributions. Two-sided *p*-values < 0.05 were considered statistically significant.

## Results

We studied 41 subjects with T2DM and 36 non-diabetic subjects (Table 1). Besides dietary advice which had been given to all diabetic subjects, metformin was taken by 5 individuals, and sulfonylurea by 6 individuals; 14 participants used both drugs. The diabetic subjects did not take other glucose lowering drugs. Additionally, anti-hypertensive drugs, particularly angiotensin-converting enzyme inhibitors, angiotensin II receptor antagonists and diuretics, alone or in combination were used by 19 T2DM subjects. None of the non-diabetic subjects used anti-hypertensive drugs. Three non-diabetic women were using estrogens. Other medications were not taken.

**Table 1:** Clinical and laboratory characteristics in 41 subjects with Type 2 diabetes mellitus (T2DM) and in 36 subjects without T2DM

	T2DM subjects (*n* = 41)	Non-diabetic subjects (*n* = 36)	*P*-value
Age (years)	60 (55–67)	51 (45–61)	< 0.001
Gender (men/women)	(19/22)	(9/27)	0.082
Systolic blood pressure (mm Hg)	142 (133–158)	128 (115–141)	< 0.001
Diastolic blood pressure (mm Hg)	86 (81–90)	78 (72–90)	0.014
BMI (kg/m^2^)	27.4 (25.5–33.0)	24.6 (22.5–26.8)	0.001
Waist (cm)	99 (90–107)	84 (76–92)	< 0.001
Glucose (mmol/l)	8.4 (7.2–10.2)	5.6 (5.0–6.2)	< 0.001
HbA1c (mmol/mol)	49 (43–58)	33 (30–37)	< 0.001
hsCRP (mg/l)	1.63 (1.03–2.87)	0.90 (0.42–2.42)	0.031
NEFA (mmol/l)	0.37 (0.29–0.45)	0.29 (0.21–0.35)	0.001
Total cholesterol (mmol/l)	5.53 (4.93–6.07)	5.46 (5.0–6.3)	0.80
Non-HDL cholesterol (mmol/l)	4.14 (3.50–4.86)	3.94 (3.39–4.53)	0.35
LDL cholesterol (mmol/l)	3.50 (2.63–3.93)	3.21 (2.78–3.84)	0.95
HDL cholesterol (mmol/l)	1.21 (1.01–1.60)	1.55 (1.32–1.82)	0.003
Triglycerides (mmol/l)	1.64 (1.21–2.16)	1.16 (0.90–1.76)	0.011
ApoB (g/l)	0.97 (0.81–1.11)	0.86 (0.73–0.96)	0.17
Apo A-1 (g/l)	1.30 (1/18–1.56)	1.46 (1.35–1.60)	0.076
ANGPTL4 (ng/ml)	5.68 (4.30–7.91)	3.42 (2.81–4.80)	< 0.001
PLTP (AU)	104.1 (98.5–113.6)	95.2 (83.4–100.0)	< 0.001

Data are medians (interquartile range). Differences between subjects with and without T2DM were determined by Mann-Whitney U tests and Chi-square tests where appropriate. *Apo*: apolipoprotein; *ANGPTL4*: Angiopoietin-like 4; *BMI*: body mass index; *HbA1c*: glycated hemoglobin; *HDL*: high density lipoproteins; *hsCRP*: high sensitivity C-reactive protein; *LDL*: low density lipoproteins; *NEFA*: non-esterified fatty acids; *PLTP*: phospholipid transfer protein; *LDL* cholesterol was calculated in 39 T2DM subjects and in 35 non-diabetic subjects

The T2DM subjects were older than the non-diabetic participants; the difference in sex distribution between T2DM and non-diabetic subjects was not significant (Table 1). Blood pressure, BMI, fasting glucose, HbA1c, hsCRP and fasting NEFA levels were higher in T2DM subjects (Table 1). Total cholesterol, non-HDL cholesterol, LDL cholesterol and apoB levels were not different between diabetic and non-diabetic subjects. Triglycerides were elevated in T2DM subjects, coinciding with lower HDL cholesterol and apoA-I. Plasma ANGPTL4 levels and PLTP activity were each higher in T2DM subjects than in non-diabetic subjects (Table 1). ANGPTL4 and PLTP activity were not different in men vs. women (ANGPTL4: 5.24 (4.13–6.01) μg/l in men and 4.35 (3.03–6.10) μg/l in women, *p* = 0.17; PLTP activity: 97.9 (92.4–103.7) AU in men and 100.2 (91.6–110.5) AU in women, *p* = 0.46).

**Fig. 1:**
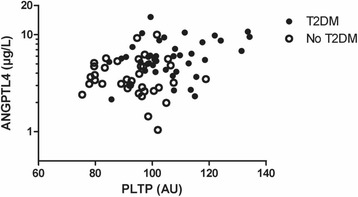
Relationship of plasma angiopoietin-like 4 (ANGPTL4) levels with phospholipid transfer protein (PLTP) activity 77 subjects (41 subjects with Type 2 diabetes mellitus (T2DM) and 36 non-diabetic subjects). Spearman correlation coefficient: 0.309, *p* = 0.006

**Fig. 2:**
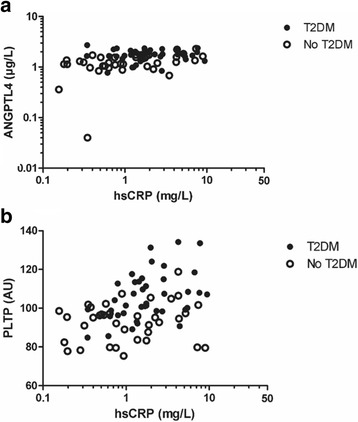
Relationship of plasma angiopoietin-like 4 (ANGPTL4) levels. **a** ANGPTL4 and phospholipid transfer protein (PLTP) activity (**b**) ANGPTL4 with high sensitivity C-reactive protein (hsCRP) in 77 subjects (41 subjects with Type 2 diabetes mellitus T2DM and 36 non-diabetic subjects. **a** Spearman correlation coefficient: 0.377, *p* = 0.001. **b** Spearman correlation coefficient: 0.373, *p* = 0.001

Univariate correlation coefficients of ANGPTL4 and PLTP activity with various clinical variables in all subjects combined are shown in Table 2. Plasma ANGPTL4 was correlated positively with PLTP activity (Fig. 1). Both ANGPTL4 and PLTP activity were correlated positively with hsCRP (Fig. 2). Furthermore, plasma ANGPTL4 and PLTP activity were both correlated positively with systolic blood pressure, BMI, glucose, hsCRP and NEFA levels. Plasma PLTP activity was also correlated positively with diastolic blood pressure. Plasma ANGPTL4 was correlated inversely with total cholesterol and LDL cholesterol, whereas PLTP activity was related positively to triglycerides and apoB, and inversely to HDL cholesterol (Table 2). Plasma ANGPTL4 was not significantly correlated with triglycerides.

**Table 2:** Univariate relationships of angiopoietin-like 4 (ANGPLT4) levels and phospholipid transfer protein (PLTP) activity with clinical and laboratory variables in 77 subjects (41 subjects with and 36 subjects without Type 2 diabetes mellitus)

	ANGPTL4 (μg/l)	PLTP (AU)
ANGPTL4		0.309**
Age (years)	0.261*	0.159
Systolic blood pressure (mm Hg)	0.211*	0.286**
Diastolic blood pressure (mm Hg)	0.137	0.268*
BMI (kg/m^2^)	0.368***	0.420***
Glucose (mmol/l)	0.341**	0.433***
HbA1c (mmol/mol)	0.447***	0.390***
hsCRP (mg/l)	0.377***	0.373***
NEFA (mmol/l)	0.411***	0.304**
Total cholesterol (mmol/l)	−0.230*	0.209
Non-HDL cholesterol (mmol/l)	−0.209	0.214
LDL cholesterol (mmol/l)	−0.255*	0.178
HDL cholesterol (mmol/l)	−0.106	−0.256*
Triglycerides (mmol/l)	0.127	0.431***
ApoB (g/l)	−0.032	0.319**
ApoA-I (g/l)	−0.054	−0.119

Spearman correlation coefficients. *Apo* apolipoprotein, *ANGPTL4* angiopoietin-like 4, *BMI* body mass index, *HbA1c* glycated hemoglobin, *HDL* high density lipoproteins, *hsCRP* high sensitivity C-reactive protein, *LDL* low density lipoproteins, *NEFA* non-esterfied fatty acids, *PLTP* phospholipid transfer protein. *LDL* cholesterol was calculated in 39 T2DM subjects and in 35 non-diabetic subjects. *p < 0.05; **p < 0.01: ***p < 0.001

Using multivariable linear regression analysis, we next tested the extent to which the relationship of plasma ANGPTL4 and PLTP activity with hsCRP was independent of NEFA, lipids (total cholesterol and triglycerides) and glycemia. ANGPTL4 remained positively associated with hsCRP when taking account of age, sex, NEFA, total cholesterol, triglycerides and the presence of diabetes (Table 3, Model A1) or alternatively of fasting glucose (Table 3, Model A2). ANGPTL4 was also independently associated with hsCRP after further adjustment for the use of metformin, sulfonylurea and antihypertensives (Model A1: β = 0.320; *p* = 0.003; Model A2: β = 0.332, *p* = 0.001). Likewise, plasma PLTP activity was positively and independently associated with hsCRP taking account of either the presence of diabetes (Table 3, Model B1) or of fasting glucose (Table 3, Model B2). The relationship of PLTP activity with hsCRP was still significant after adjustment for the use of metformin, sulfonylurea and antihypertensives (Model A1: β = 0.224; *p* = 0.044; Model A2: β = 0.266, *p* = 0.016). In subsidiary analyses with BMI in addition to age, sex, the presence of diabetes, NEFA, total cholesterol and triglycerides as dependent variables, plasma PLTP activity but not ANGPTL4 was independently associated with BMI (β = 0.239, *p* = 0.05 and β = 0.137, *p* = 0.23, respectively; data not shown).

**Table 3:** Multivariable regression analyses showing independent relationships of angiopoietin-like 4 (ANGPTL4) levels and phospholipid transfer protein (PLTP) activity with high sensitivity C-reactive protein (hsCRP), glycemia and plasma lipids in 77 subjects (41 subjects with and 36 subjects without Type 2 diabetes mellitus)

	Model A 1 ANGPTL4		Model A 2 ANGPTL4		Model B 1 PLTP activity		Model B 2 PLTP activity	
	β	*p*-value	β	*p*-value	β	*p*-value	β	*p*-value
Age	0.173	0.12	0.209	0.065	0.094	0.43	0.119	0.33
Sex (men vs. women)	0.226	0.043	0.263	0.014	−0.135	0.25	−0.057	0.62
T2DM	0.088	0.48			0.376	0.006		
Glucose			−0.081	0.51			0.276	0.034
hsCRP	0.327	0.002	0.315	0.003	0.229	0.037	0.299	0.034
NEFA	0.360	0.002	0.374	0.001	0.020	0.87	0.105	0.37
Total cholesterol	−0.284	0.009	−0.308	0.005	0.014	0.90	0.025	0.83
Triglycerides	0.081	0.47		0.105	0.39	0.10	0.166	0.21

Models A: ANGPTL4 as dependent variable

Models B: PLTP activity as dependent variable

Models 1: including Type 2 diabetes mellitus (T2DM) as independent variable

Models 2: including fasting glucose as independent variable

β: standardized regression coefficient, *NEFA* non-esterified fatty acids, *T2DM* Type 2 diabetes mellitus. ANGPTL4, hsCRP and triglycerides are log_e_ transformed

In view of the observation that plasma ANGPTL4 and PLTP activity were positively correlated with each other in univariate analysis, and that both ANGPTL4 and PLTP activity were independently associated with hsCRP, we next sought to determine whether the relationship between plasma ANGPTL4 and PLTP activity was independent of plasma glucose, total cholesterol and triglycerides, In age- and sex-adjusted multivariable linear regression analysis, plasma ANGPTL4 was independently associated with PLTP activity (Table 4, Model A). This relationship was essentially unaltered after further adjustement for the use of metformin, sulfonylurea and antihypertensives (β = 0.226, *p* = 0.058). Notably, when hsCRP was included in the analysis, the relationship between plasma ANGPTL4 and PLTP activity disappeared (Table 4, Model B). This finding is consistent with the possibility that the relationship of plasma ANGPTL4 with PLTP activity is at least in part explained by their associations with hsCRP.

**Table 4:** Multivariable regression analyses showing relationships between angiopoietin-like 4 (ANGPTL4) levels and phospholipid transfer protein (PLTP) activity with high sensitivity C-reactive protein (hsCRP), glycemia and plasma lipids in 77 subjects (41 subjects with and 36 subjects without Type 2 diabetes mellitus)

	Model A		Model B	
	β	*p*-value	β	*p*-value
Age	0.139	0.23	0.209	0.065
Sex (men vs. women)	0.239	0.033	0.263	0.014
Glucose	−0.069	0.59	−0.081	0.51
NEFA	0.378	0.001	0.374	0.001
Total cholesterol	−0.301	0.010	−0.308	0.005
Triglycerides	0.133	0.30	0.105	0.39
PLTP activity	0.244	0.032	0.131	0.25
hsCRP			0.315	0.003

Model A: relationship of plasma ANGPTL4 with PLTP activity with hsCRP not being included as dependent variable

Model B: relationship of plasma ANGPTL4 with PLTP activity with hsCRP being included as dependent variable

β: standardized regression coefficient, *NEFA*: non-esterified fatty acids, *T2DM* Type 2 diabetes mellitus. ANGPTL4, hsCRP and triglycerides are log_e_ transformed

## Discussion

The main novel finding of the present study is that the plasma ANGPTL4 concentration is positively correlated with PLTP activity. This relationship remained present when taking account of age, sex, fasting glucose, NEFA and lipid levels. Notably, ANGPTL4 was no longer associated with PLTP activity after further adjustment for hsCRP, a marker of low-grade chronic inflammation. Since our study also shows that ANGPTL4 and PLTP activity are each independently associated with hsCRP, these findings are consistent with the hypothesis that enhanced low-grade inflammation may result in increased circulating levels of both ANGTPL4 and PLTP activity. This would explain at least in part the positive relationship between ANGTPL4 and PLTP. In addition, given the different mechanisms whereby ANGPTL4 and PLTP act on triglyceride metabolism [1–3, 11–15], it is plausible to postulate that ANGPTL4 and PLTP may act together to elevate circulating triglycerides under pro-inflammatory circumstances.

In agreement with several earlier reports [10, 20], plasma ANGPTL4 was elevated in T2DM subjects. However, neither the presence of T2DM nor the fasting glucose concentration predicted plasma ANGPTL4 in age- and sex-adjusted multivariable linear regression analysis in which we also accounted for hsCRP, NEFA, total cholesterol and triglycerides. hsCRP and NEFA levels were elevated in T2DM, and in view of their independent relationships with plasma ANGPTL4, it is likely that these variables jointly contributed to the higher ANGPTL4 levels as presently observed in T2DM. On the other hand in fully adjusted analysis, plasma PLTP activity remained associated with T2DM or alternatively with fasting glucose, in keeping with other data [11–13, 23, 27]. In addition, although both plasma ANGPTL4 and PLTP activity were expectedly correlated with obesity and NEFA, ANGPTL4 was inversely correlated with total cholesterol and LDL cholesterol, whereas PLTP activity was significantly correlated with triglycerides [8, 9, 11, 12]. Furthermore, the production of ANGTPL4 is stimulated by PPARγ activation in vitro [28], whereas plasma PLTP activity was found to change little in response to the PPARγ agonist pioglitazone in vivo in humans [29]. Thus, the interrelationship between plasma ANGPTL4 and PLTP activity, even after adjustment for dysglycemia, NEFA and lipid levels, is remarkable.

ANGPTL4 is upregulated by lipopolysaccharide (LPS) which likely involves the Toll-like receptor 4 (TRL 4) [10, 30]. Interestingly, PLTP shares homology with LPS [12, 31] but while PLTP increases in response to acute inflammation, it may decrease after LPS administration [24]. In addition, our unpublished data indicate that the level of Angptl4 mRNA in rat hepatocytes is markedly increased by interleukin-1β (IL-1β) and tumor necrosis factor α (TNFα). On the other hand, the ability of interleukin-6 (IL-6) to induce TNFα is abolished in PLTP knockout mice [32], raising the possibility that TNFα could be involved in interrelation between ANGPTL4 and PLTP. We used hsCRP as a marker of enhanced low-grade chronic inflammation, but it seems unlikely that CRP plays a major direct role in regulating ANGPTL4 and PLTP. Obviously however, given the complex and yet to be more precisely delineated processes by which inflammatory stimuli affect ANGTPL4 and PLTP regulation, the mechanisms responsible for the association of a pro-inflammatory state with both plasma ANGPTL4 and PLTP activity await further study.

Loss of function mutations in ANGPTL4 and genetic variations in PLTP that attenuate its plasma activity have been associated with an attenuated risk of coronary artery disease, emphasizing the relevance of ANGTP4- and PLTP-mediated processes on the development of atherosclerosis [33, 34]. Based in the present findings, we postulate that yet to be more precisely delineated inflammatory pathways may give rise to increased plasma levels of ANGPTL4 and PLTP activity, conceivably contributing to joint effects on triglyceride elevations. Thus, in the context of low-grade chronic inflammation, ANGPTL4 and PLTP gene-environment interactions could aggravate abnormalities in NEFA and triglyceride metabolism.

Several other methodological issues of the present study need consideration. The ANGPTL4 ELISA that we used measures the full length protein and the C-terminal truncated fragment but does not detect the N-terminal truncated fragment. The N-terminal but not the C-terminal fragment carries LPL inhibitory activity. Concerning PLTP, its activity as reported here reflects the level of active PLTP [25, 26]. We preferred to use this type of activity assay, which is independent of the endogenous lipoproteins present in the individual patient samples, because PLTP circulates in an active and inactive form. Consequently, PLTP activity and mass levels are not closely related [35–37]. Furthermore, we excluded diabetic subjects using insulin and lipid lowering drugs in order to obviate confounding due to effects on glucose and lipid metabolism. As a result, we mainly included T2DM subjects with rather mild hyperglycemia and dyslipidemia which would limit the generalizability of our findings. Also, in view of the cross-sectional design of the current study, it should be stressed that cause-effect relationships cannot be established with certainty. Indeed, PLTP is involved in the innate immune system, and has been implicated to causally modulate inflammatory processes [31, 32]. Finally, we regard the present findings as preliminary given the limited numbers of diabetic and non-diabetic participants.

## Conclusion

In conclusion, plasma ANGPTL4 and PLTP activity are interrelated, which may in part be attributable to a stimulatory influence of low grade inflammation on these two protein fators. A pro-inflammatory state could affect triglyceride metabolism via concerted effects on ANGPTL4 and PLTP.

## Abbreviations

ANGPTL: Angiopoietin like proteins
apoA-I: Apolipoprotein A-I
apoB: Apolipoprotein B
BMI: Body mass index
HDL: High density lipoproteins
hsCRP: High sensitivity C-reactive protein
IL-1β: Interleukin-1β
IL-6: Interleukin-6
NEFA: Non-esterified fatty acids
PLTP: Phospholipid transfer protein
PPARs: Peroxisome proliferator-activated receptors
T2DM: Type 2 diabetes mellitus
TNFα: Tumor necrosis factor α
VLDL: Very low density lipoproteins
